# The association of HLA-G polymorphism with oral and genital HPV infection in men

**DOI:** 10.1007/s10096-021-04362-8

**Published:** 2021-10-25

**Authors:** Nelli T. Suominen, Anna J. Jaakola, Michel Roger, Marie-Claude Faucher, Kari J. Syrjänen, Seija E. Grénman, Stina M. Syrjänen, Karolina Louvanto

**Affiliations:** 1 Department of Obstetrics and Gynecology, Turku University Hospital, University of Turku, Kiinamyllynkatu 4-8, 20521 Turku, Finland; 2 Department of Obstetrics and Gynecology, Kymenlaakso Central Hospital, Kotkantie 41, 48210 Kotka, Finland; 3 Centre de Recherche, Centre Hospitalier de l’Université de Montréal, 900, rue Saint-Denis, Pavillon R, Montréal, Québec H2X 0A9 Canada; 4 Département de Microbiologie, Infectiologie, Immunologie, Université de Montréal, Roger-Gaudry Building, 2900 Edouard Montpetit Blvd, Montreal, QC H3T 1J4 Canada; 5 Department of Clinical Research, Biohit Oyj, Laippatie 1, 00880 Helsinki, Finland; 6 Department of Oral Pathology and Radiology, University of Turku, Lemminkäisenkatu 2, 20520 Turku, Finland; 7 Department of Obstetrics and Gynecology, Tampere University Hospital, Tampere University, Finn-Medi 1, Biokatu 6, 33100 Tampere, Finland

**Keywords:** HLA-G, Human papillomavirus, HPV, Men, Oral, Genital

## Abstract

The host genetic factors that influence the natural history of human papillomavirus (HPV) infection in men are not well known. Our aim was to evaluate the role of human leukocyte antigen (HLA)-G polymorphism in oral and genital HPV infection in men. Altogether, 130 men from the Finnish Family HPV Study, with a 6-year follow-up, were included in the analyses. HLA-G alleles were tested by direct sequencing. Oral, urethral, and semen samples were collected and analyzed for 24 different HPV genotypes. Unconditional logistic regression was used to determine associations between HLA-G alleles and genotypes with HPV infection and its outcomes. Overall, eight different HLA-G alleles were identified with 15 different HLA-G genotype combinations. The most common HLA-G allele among the men was G*01:01:01 (86.2%, *n* = 112) followed by G*01:01:02 (36.2%, *n* = 47). Allele G*01:01:02 showed to be protective against any- and high-risk (HR) oral HPV (OR range of 0.20–0.24, 95% CI range of 0.06–0.85). Men having allele G*01:01:01 showed a reduced risk for incident (OR 0.30, 95% CI 0.11–0.84) and persistent (OR 0.24, 95% CI 0.08–0.69) oral infections. Allele G*01:01:03 was associated with increased risk for urethral HR-HPV infections (OR 4.94, 95% CI 1.34–18.27). Among self-reported demographic data, genotype G*01:01:01/01:01:03 was associated with an increased risk for oral warts (OR 8.00, 95% CI 1.23–51.89) and allele G*01:03:01 increased the risk of pollen and/or animal allergy (OR 13.59, 95% CI 1.57–117.25). To conclude, HLA-G polymorphism in men largely impacts the outcome of an oral HPV infection and seems to associate with self-reported allergies.

**Supplementary Information:**

The online version contains supplementary material available at 10.1007/s10096-021-04362-8.

## Introduction

Human papillomavirus (HPV) infections are typically asymptomatic, transient, and cleared by the host immune system. However, persistent infection with certain high-risk (HR) HPV types is associated with HPV-related cancers, most importantly cervical cancer which is the fourth most common cancer in women worldwide [1]. In men, anal, penile, and oropharyngeal cancers are attributable to HPV infections [2]. In 2018, 92,887 patients were diagnosed with oropharyngeal carcinoma globally [1]. Oropharyngeal carcinoma is four times more prevalent in men than in women [1]. Around 30% of oropharyngeal cancers, 29,000 cases per year, are caused by HPV; 24,000 of those occur in males [2].

Genital HPV prevalence in men ranges widely depending on the population and type of sampling sites, and at its highest is shown to be 72.9% but more usually ≥ 20% [3]. Compared to women, the peak prevalence of male genital HPV infection is suggested to occur at a slightly older age and its prevalence remains more stable during an individual’s lifetime [4]. However, the natural history of HPV is much less studied in men than in women. Sexual activity, uncircumcision, and smoking are associated with the higher possibility of acquiring HPV infection [5]. Men’s seminal HPV infection has also been linked to male infertility [6]. As well as behavioral risk factors have been identified, certain viral and hosts genetic as immunological co-factors are known to affect the natural history of HPV infection. It is supposed that immunological mechanisms play an important role in acquiring and clearing both genital and oral HPV infection.

The human leukocyte antigen (HLA)-G has been proposed as a possible immunological co-factor in the pathogenesis of HPV as in other viral infections. HLA-G, as a member of the non-classical human leukocyte antigen (HLA) class Ib, acts as a negative regulator of immune responses through interactions between natural killer (NK), T, and antigen-presenting cells [7]. Compared to the classical class I HLA (HLA-A, HLA-B, HLA-C), HLA-G has a low allelic polymorphism and is very tissue specific [8]. HLA-G has been found to be expressed under pathological conditions like cancer, autoimmune disease, tissue transplantation, and viral infections [8–10]. The host factors that influence the natural history of HPV remain mostly unknown, and existing information is mainly based on female data [11]. The aim of our study was to evaluate the association of HLA-G polymorphism with oral and genital HPV infections in men.

## Methods

### Finnish Family HPV Study

The Finnish Family HPV (FFHPV) Study is a longitudinal cohort followed up at Turku University Hospital, University of Turku, Finland. The study design was to evaluate the dynamics of HPV infection among family members: mothers, fathers, and their offspring as described previously [12, 13]. A total of 329 families with 329 women, 131 men, and their 331 newborns were enrolled at baseline (36 weeks of pregnancy) between years 1998 and 2002 and were followed up for 6 years. The cohort represents Caucasian origin as the Finnish population has the same ethnic background.

### Samples

In the present study, the previously published data on HPV infection in men was utilized [14–18]. At enrolment, semen, urethral, and oral samples were collected for HPV testing as previously described [15, 17]. The HPV data of semen, urethral, and oral baseline samples were available as given in Table S1 (online resource). Follow-up samples for HPV genotyping were available solely from the oral cavity. During the 6-year follow-up, oral brush samples were collected at baseline and approximately at 2, 6, 12, 24, 36, and 77 months [17].

HPV DNA was extracted from samples by using the high salt method as described previously [19]. HPV amplification was done by nested PCR with MY09/MY11 primers first, and second with GP05 and biotinlated-GP06 [20]. HPV genotyping was performed by the Multimetrix kit (Multimetrix, Progen Biotechnik GmbH, Heidelberg, Germany) identifying 24 different low-risk (LR) and high-risk (HR) HPV genotypes (LR genotypes: 6, 11, 42, 43, 44 and HR genotypes: 16, 18, 26, 31, 33, 35, 39, 45, 51, 52, 53, 56, 58, 59, 66, 68, 70, 73, 82).

### HLA-G determination

DNA for HLA-G typing was extracted from the frozen whole blood samples by using the MagNAPure 96 System (Roche). Determination of HLA-G alleles was done by direct DNA sequencing exploring exons 2–4 (1718 bp) of HLA-G gene regions as described previously [21].

### Background information

The demographic data of the men were collected by a structured questionnaire at baseline. The mean age of the men was 28.9 years. The baseline demographic data have been reported in detail previously [15]. The questionnaire included a self-reported history of infertility, previous chlamydia and mumps infections, asthma, allergy, and atopy. Men were also questioned about their history of HPV-related symptoms and signs of genital, skin, and oral warts.

### Statistical analysis

All statistical analyses were run by using STATA SE15.1 (StataCorp, College Station, TX, USA). The present study included overall 130 men, who had both completed HLA-G allele testing and had a ≥ 1 oral HPV genotyping result available. Baseline samples for two men were missing, leaving 128 men with HPV result of their semen, urethra, and oral baseline samples for analyses. Only those HLA-G alleles and genotypes that were ≥ 3% prevalent among men were included in the analyses. HPV species were classified as either LR or HR groups [22]. These groups were used for comparisons to determine the association between HLA-G (alleles and genotypes) and semen, urethral, and oral HPV positivity at baseline. Odds ratios (OR) and their 95% confidence intervals (CI) were calculated by using unconditional logistic regression. To estimate the effect of each allele, subjects who tested positive for an allele (homozygous or heterozygous) were compared to those who tested negative for that allele.

Four oral HPV outcome variables were defined: always HPV negative, incident HPV, clearance, and persistence. Always HPV negative denoted men who tested negative for HPV at baseline and every follow-up visit. Incident HPV included men who tested negative at enrolment and then positive at some point during follow-up. Clearance included men tested HPV positive at baseline or at some point in the follow-up, then turned HPV negative before the end of the follow-up and remained negative until the end of follow-up. Persistence was defined whenever a man tested positive for the same HPV genotype with two or more consecutive visits. This definition also included the men who tested consecutively positive with the same HPV genotypes as part of a multiple infection.

Unconditional logistic regression was used to determine associations (OR) between HLA-G: alleles as genotypes, and oral HPV infection outcomes. Those men in whom the allele was absent were used as reference. Associations of possible risk factors with HLA-G alleles and genotypes were analyzed by using unconditional logistic regression. All the statistical tests were performed two-sided and the value *p* < 0.05 was regarded as statistically significant.

## Results

**Fig. 1:**
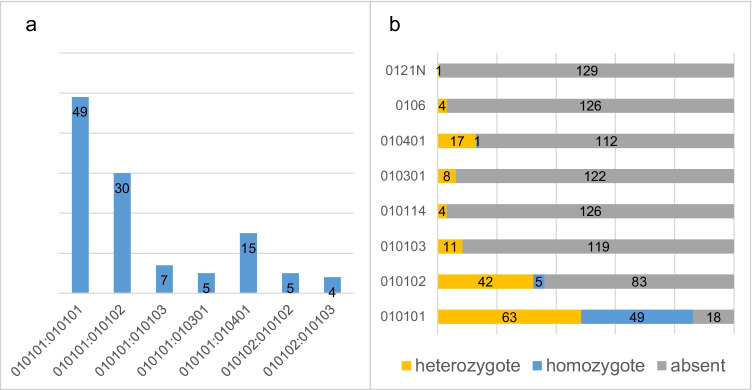
HLA-G genotype (**a**) and allele (**b**) distribution among the 130 men from the Finnish Family HPV Study. Those alleles and genotypes that were ≥ 3% prevalent were included. Different HLA-G genotypes (**a**) are marked on the *x*-axis, while the number of men with certain genotype is presented on the *y*-axis. Stacked bar columns represent the distribution of different alleles (**b**) (absent = missing allele, heterozygote = having one allele, homozygote = having two alleles)

Overall, eight different HLA-G alleles with 15 different HLA-G genotype combinations were identified. Altogether, seven different HLA-G alleles and genotypes (≥ 3% prevalent) were included in the analyses. The allele and genotype distributions are shown in Fig. 1. The most common HLA-G allele was the wild-type G*01:01:01 (86.2%, *n* = 112) followed by G*01:01:02 (36.2%, *n* = 47). The most common genotype found was G*01:01:01/01:01:01 (37.7%, *n* = 49) followed by G*01:01:01/01:01:02 (23.1%, *n* = 30), respectively.

At baseline, the frequency (%) of different HPV genotypes in semen (*n* = 86), urethral (*n* = 122) and oral (*n* = 128) samples is shown in Table S1. The HPV DNA was more frequently detected in semen samples (31.4%, 27/86) than in urethral (22.1%, 27/122) or oral (18.8%, 24/128) samples. HR-HPV was found in 26.7% (23/86), 16.4% (20/122), and 16.4% (21/128) in semen, urethral, and oral samples, respectively, whereas LR-HPV accounted for 10.5% (9/86), 7.4% (9/122), and 3.1% (4/128) of semen, urethral, and oral samples, respectively. For the HPV genotype distribution, multiple-type infections were sorted out as individual HPV genotypes. Two or more different HPV genotypes detected in the same sample were defined as a multiple infection which accounted for 6/86, 6/122, and 4/128 of semen, urethral, and oral baseline samples, respectively.

To evaluate the influence of HLA-G alleles and genotypes on HPV baseline prevalence in men, different alleles and genotypes were compared to the frequency of any HPV, LR, and HR positivity in all three baseline-tested anatomical samples: semen, urethra, and oral, as seen in Table 1. Allele G*01:01:02 was protective for any oral HPV infection with an OR of 0.20 (95% CI 0.06–0.72), and also for oral HR-HPV infections, with an OR of 0.24 (95% CI 0.07–0.85). Allele G*01:01:03 was associated with an increased risk for urethral HR-HPV infections (OR 4.94, 95% CI 1.34–18.27). As regards the HLA-G genotypes, no significant associations were found between HLA-G genotypes and HPV positivity at the evaluated sites.

**Table 1:** Associations of different HLA-G alleles and genotypes with the semen, urethral, and oral HPV positivity among 128 men of the Finnish Family HPV Study at baseline. Significant results bolded. *p*-value <0.05

HLA-G	Semen OR (95% CI)			Urethra OR (95% CI)			Oral OR (95% CI)		
	any HPV + (*n* = 27)	LR (*n* = 9)	HR (*n* = 23)	any HPV + (*n* = 27)	LR (*n* = 9)	HR (*n* = 20)	any HPV + (*n* = 24)	LR (*n* = 4)	HR (*n* = 21)
Alleles^a,b^									
*01:01:01	1.83 (0.47–7.20)	0.80 (0.15–4.40)	2.41 (0.49–11.82)	0.70 (0.22–2.17)	1.27 (0.15–10.99)	0.63 (0.18–2.20)	4.49 (0.57–35.56)	NA	3.91 (0.49–31.11)
*01:01:02	0.73 (0.28–1.89)	2.92 (0.66–12.81)	0.51 (0.18–1.49)	1.06 (0.43–2.56)	0.90 (0.21–3.82)	1.20 (0.45–3.21)	**0.20 (0.06**–**0.72)**	NA	**0.24 (0.07**–**0.85)**
*01:01:03	0.25 (0.03–2.07)	0.80 (0.09–7.25)	0.29 (0.03–2.46)	3.37 (0.94–12.07)	NA	**4.94 (1.34**–**18.27)**	1.71 (0.42–7.01)	NA	2.00 (0.48–8.27)
*01:01:14	1.10 (0.10–12.64)	NA	1.30 (0.11–15.02)	3.72 (0.50–27.74)	5.81 (0.47–71.29)	2.45 (0.21–28.38)	NA	NA	NA
*01:03:01	NA	NA	NA	0.48 (0.057–4.11)	NA	0.66 (0.077–5.70)	NA	NA	NA
*01:04:01	3.13 (0.77–12.73)	NA	3.82 (0.92–15.78)	0.46 (0.098–2.18)	0.72 (0.08–6.24)	0.30 (0.038–2.46)	2.56 (0.85–7.70)	2.56 (0.25–26.58)	2.40 (0.74–7.72)
*01:06	2.28 (0.30–17.11)	3.56 (0.29–43.92)	2.71 (0.36–20.52)	1.18 (0.12–11.82)	3.83 (0.36–41.24)	NA	1.46 (0.15–14.72)	11.22 (0.89–141.98)	NA
Genotypes^b^									
*01:01:01/01:01:01	0.99 (0.39–2.54)	0.48 (0.09–2.52)	1.08 (0.40–2.91)	0.78 (0.32–1.93)	0.78 (0.18–3.33)	0.84 (0.31–2.31)	1.89 (0.77–4.63)	1.89 (0.26–13.97)	2.08 (0.81–5.36)
*01:01:01/01:01:02	1.24 (0.43–3.57)	2.83 (0.66–12.09)	0.98 (0.31–3.16)	1.01 (0.36–2.82)	1.01 (0.19–5.21)	1.17 (0.38–3.61)	0.26 (0.06–1.18)	NA	0.30 (0.07–1.37)
*01:01:01/01:01:03	NA	NA	NA	2.84 (0.60–13.57)	NA	4.01 (0.82–19.57)	3.57 (0.74–17.15)	NA	4.17 (0.86–20.21)
*01:01:01/01:04:01	3.25 (0.67–15.66)	NA	3.93 (0.81–19.17)	0.27 (0.03–2.14)	0.86 (0.10–7.54)	NA	2.47 (0.76–8.06)	3.13 (0.30–33.03)	2.21 (0.62–7.87)
*01:01:02/01:01:02	NA	NA	NA	0.88 (0.09–8.17)	2.84 (0.28–28.58)	NA	NA	NA	NA
*01:01:02/01:01:03	0.72 (0.07–7.24)	2.33 (0.22–25.24)	0.85 (0.08–8.60)	3.72 (0.50–27.74)	NA	5.17 (0.68–39.10)	NA	NA	NA
*01:01:01/01:03:01	NA	NA	NA	0.88 (0.09–8.17)	NA	1.20 (0.13–11.32)	NA	NA	NA

^a^Being homozygote or heterozygote for the HLA-G gene

^b^Only those alleles and genotypes that where ≥ 3% prevalent among men were included in the analyses

The associations of HLA-G (alleles and genotypes) with oral HPV outcomes (always HPV negative, incident HPV, HPV clearance, and HPV persistence) are shown in Table 2. Men having allele G*01:01:01 seemed to have a lower risk for incident oral HPV infection (OR 0.30, 95% CI 0.11–0.84). Interestingly, allele G*01:01:01 was also associated with a lower risk for persistent oral infections (OR 0.24, 95% CI 0.08–0.69). No significant associations between any HLA-G genotypes and the oral HPV outcomes were observed.

**Table 2:** Associations of HLA-G alleles and genotypes with oral HPV infection outcomes among 130 men of the Finnish Family HPV Study. Significant results bolded. *p*-value <0.05

HLA-G	Always HPV OR (95% CI)	Incidence OR (95% CI)	Clearance OR (95% CI)	Persistence OR (95% CI)
Alleles^a,b^				
*01:01:01	1.50 (0.53–4.28)	**0.30 (0.11**–**0.84)**	1.38 (0.36–5.20)	**0.24 (0.08**–**0.69)**
*01:01:02	1.07 (0.52–2.20)	1.77 (0.85–3.71)	0.54 (0.19–1.57)	2.06 (0.86–4.92)
*01:01:03	1.78 (0.51–6.15)	0.37 (0.08–1.77)	0.55 (0.09–3.57)	1.57 (0.38–6.36)
*01:01:14	NA	NA	1.16 (0.11–11.89)	NA
*01:03:01	2.48 (0.57–10.87)	1.06 (0.24–4.66)	NA	1.36 (0.26–7.17)
*01:04:01	1.15 (0.42–3.13)	0.46 (0.14–1.48)	3.98 (0.47–33.64)	0.77 (0.21–2.90)
*01:06	NA	5.59 (0.56–55.35)	0.36 (0.05–2.75)	4.25 (0.57–31.71)
Genotypes^b^				
*01:01:01/01:01:01	0.83 (0.40–1.72)	0.78 (0.37–1.65)	1.26 (0.43–3.68)	0.54 (0.21–1.41)
*01:01:01/01:01:02	1.31 (0.58–2.98)	1.03 (0.44–2.40)	0.63 (0.18–2.17)	1.00 (0.36–2.77)
*01:01:01/01:01:03	1.95 (0.42–9.08)	NA	0.76 (0.07–8.88)	1.65 (0.30–9.03)
*01:01:01/01:04:01	1.27 (0.43–3.73)	0.40 (0.11–1.51)	2.96 (0.34–25.70)	0.26 (0.03–2.05)
*01:01:02/01:01:02	0.94 (0.15–5.80)	2.76 (0.44–17.15)	0.76 (0.07–8.88)	2.81 (0.44–17.73)
*01:01:02/01:01:03	1.42 (0.19–10.43)	1.80 (0.25–13.21)	0.37 (0.02–6.26)	1.35 (0.13–13.50)
*01:01:01/01:03:01	6.00 (0.65–55.26)	0.43 (0.05–3.96)	NA	NA

^a^Being homozygote or heterozygote for the HLA-G gene

^b^Only those alleles and genotypes that where ≥ 3% prevalent among men were included in the analyses

The association of the self-reported demographic data of the men and their association with HLA-G alleles and genotypes are given in Table 3. Allele G*01:03:01 was significantly associated with an increased risk for an allergy (OR 13.59, 95% CI 1.57–117.25). A total of 33.9% (40/118) of men reported allergic symptoms for pollen and/or animals. Food allergies were excluded from this variable. Among the genotype-level analyses, 6.3% (7/112) of men reported a history of oral warts for which genotype G*01:01:01/01:01:03 appeared to be a significant risk factor (OR of 8.00, 95% CI 1.23–51.89). In allele-specific analyses, G*01:01:03 showed a high OR of 4.85 (95% CI 0.81–29.09) for oral warts but due to a wide CI, it did not reach statistical significance.

**Table 3:** Association of HLA-G alleles and genotypes with the demographic data of 130 men collected by questionnaire at baseline. Significant results bolded. *p*-value <0.05

	HLA-G allele^a,b^						
	*01:01:01	*01:01:02	*01:01:03	*01:01:14	*01:03:01	*01:04:01	*01:06
Infertility	0.51 (0.10–2.68)	2.50 (0.63–9.87)	3.68 (0.65–20.71)	4.50 (0.42–48.35)	6.06 (0.99–36.99)	NA	NA
History of chlamydia infection	1.38 (0.30–6.28)	0.74 (0.21–2.57)	0.58 (0.06–6.06)	NA	0.90 (0.08–10.77)	9.33 (0.94–92.47)	0.90 (0.08–10.77)
History of genital warts	0.50 (0.14–1.77)	1.55 (0.57–4.25)	1.26 (0.25–6.48)	5.41 (0.71–41.09)	0.81 (0.09–7.19)	1.07 (0.28–4.16)	1.69 (0.17–17.13)
History of oral warts	0.35 (0.06–2.01)	2.78 (0.59–13.14)	4.85 (0.81–29.09)	NA	2.75 (0.28–26.66)	NA	5.67 (0.51–62.98)
History of skin warts	2.64 (0.79–8.86)	1.08 (0.50–2.35)	1.85 (0.49–6.95)	0.38 (0.04–3.75)	0.45 (0.08–2.41)	0.79 (0.28–2.25)	NA
History of mumps	0.59 (0.17–2.01)	1.35 (0.60–3.05)	1.07 (0.24–4.71)	0.63 (0.09–4.62)	0.45 (0.10–2.14)	0.79 (0.27–2.31)	NA
Asthma	NA	NA	3.93 (0.37–41.72)	NA	NA	NA	NA
Allergy	0.39 (0.13–1.18)	1.27 (0.57–2.82)	2.09 (0.57–7.68)	0.64 (0.06–6.37)	**13.59 (1.57–117.25)**	0.37 (0.10–1.38)	NA
Atopy	0.40 (0.07–2.20)	0.61 (0.12–3.15)	4.25 (0.74–24.57)	5.10 (0.47–55.54)	NA	2.11 (0.39–11.45)	NA
	HLA-G genotypes^b^						
	*01:01:01/01:01:01	*01:01:01/01:01:02	*01:01:01/01:01:03	*01:01:01/01:04:01	*01:01:02/01:01:02	*01:01:02/01:01:03	*01:01:01/01:03:01
Infertility	0.19 (0.02–1.58)	1.81 (0.42–7.79)	6.06 (0.99–36.99)	NA	NA	NA	4.50 (0.42–48.35)
History of chlamydia infection	1.58 (0.43–5.77)	0.73 (0.16–3.30)	0.90 (0.08–10.77)	NA	NA	NA	NA
History of genital warts	0.55 (0.18–1.66)	0.99 (0.29–3.30)	2.09 (0.38–11.69)	0.73 (0.15–3.55)	1.69 (0.17–17.13)	NA	NA
History of oral warts	0.26 (0.03–2.24)	1.51 (0.27–8.31)	**8.00 (1.23–51.89)**	NA	NA	NA	NA
History of skin warts	1.29 (0.61–2.74)	1.87 (0.75–4.64)	0.87 (0.19–4.07)	1.03 (0.35–3.05)	0.38 (0.04–3.75)	NA	1.18 (0.16–8.65)
History of mumps	0.93 (0.43–2.01)	1.17 (0.47–2.95)	1.29 (0.23–7.37)	0.60 (0.19–1.84)	1.95 (0.20–19.41)	0.63 (0.04–10.38)	0.63 (0.09–4.62)
Asthma	5.21 (0.53–51.74)	NA	6.06 (0.55–67.27)	NA	NA	NA	NA
Allergy	0.96 (0.44–2.11)	0.83 (0.33–2.13)	1.50 (0.32–7.05)	0.26 (0.06–1.23)	2.00 (0.27–14.75)	4.05 (0.36–46.11)	6.24 (0.63–62.08)
Atopy	0.54 (0.10–2.80)	0.46 (0.05–3.93)	2.48 (0.26–23.52)	0.98 (0.11–8.57)	NA	7.71 (0.62–95.80)	NA

^a^Being homozygote or heterozygote for the HLA-G gene

^b^Only those alleles and genotypes that where ≥ 3% prevalent among men were included in the analyses

## Discussion

To our knowledge, this is the first study reporting on the association of HLA-G polymorphism and HPV infection in men. To date, most studies have focused on HLA-G polymorphism and HPV-related diseases in women [21, 23–29]. The few studies in which male participants were included have mainly focused on exploring HLA-G polymorphism in the HLA-G 3′ untranslated region (UTR) and disclosed an association with other viral infections, such as HCV [30], HBV [31, 32], and HIV [33, 34]. The data evaluating HLA-G specifically among men is sparse. We found one previous study reporting the influence of HLA-G 3^′^UTR polymorphisms in prostate cancer susceptibility [35].

The male cohort of the present study represents a Finnish, Caucasian, male population which has a quite restricted and homogenous gene pool due to historical isolation. The relatively low number of different HLA-G alleles observed is in line with our recent observations of the women in the same cohort [29] and is explained by the cohort characteristic mentioned above. The wild-type G*01:01:01 allele was the most common allele found in these men, similar to the spouses of these males [29] and also in the studies among Brazilian [36] and Canadian [37] populations.

Our main finding was that the presence of wild-type *G01:01:01 allele in men was shown to be protective for incident and persistent oral HPV infections. Moreover, the *G01:01:02 allele was associated with a lower risk for any and HR oral HPV infections. Interestingly, part of our results is contradictory to that reported in studies on HLA-G polymorphism and HPV infection among women, which are focused on genital infections [21, 24, 36, 37]. Consistent with our results, Metcalfe et al. reported that G*01:01:02 allele was associated with a lower risk of genital HPV infection among 548 Inuit women [24]. Intriguingly, G*01:01:02 allele has also been found to have protecting function for cervical intraepithelial lesions (CIN) among HIV-positive pregnant women [36].

We found that G*01:01:01 allele decreased the risk for incident and persistent oral HPV infection by any viral type. In contrast, Metcalfe et al. reported that G*01:01:01 allele was associated with an increased risk of genital LR-HPV infection [24]. Ferguson et al. reported that the heterozygotic form of the wild-type G*01:01:01 allele was associated with a lower risk of cervical cancer [37]. Since persistent HPV infection is mandatory for HPV-induced oral malignant transformation, one can speculate that G*01:01:01 might be a protective host factor against persistent HPV infection in both genital and oral sites.

In addition, we found that allele G*01:03:01 in men was significantly associated with an increased risk for allergies to pollen and/or animals. Several studies have shown that HLA-G is associated with allergic diseases as reviewed by Murcada et al. [38]. In general, HLA-G is a tolerance-inducing molecule, but it is also a stimulus for T helper 2 cell (Th2) responses and regulatory T cell (Treg) activation. Allergic diseases are driven by a Th2-polarized inflammation and allergic patients display a defect in Treg cells which may be restored by specific immunotherapy [39, 40]. Thus, one could speculate that immunomodulation by HLA-G can increase the risk for HPV infection as has recently been shown in the case with an HCV infection [41].

The main limitation of our study was the rather low number of men with uncommon HLA-G alleles. This might have interfered with the detection rate of significant associations between HLA-G and HPV infections in men. The demographic data was also self-reported, which can create some bias especially with the self-reported wart diagnosis. We had no similar follow-up data for genital samples as we had for oral samples. Thus, the evaluation of the role of HLA-G in HPV infection outcomes had to be restricted to only the oral site. The strength of the present study is a representative cohort with the same marital status and ethnic background which also reflected the genotype distribution that we observed. Although the follow-up time was relatively long, 76.2% (99/130) of the men completed the 3-year follow-up and 42.3% (55/130) the full 6-year follow up with oral samples available from each follow-up visit. To conclude, this is the first study to disclose that HLA-G polymorphism seems to play a role in oral HPV infections in males. Thus, the role of HLA-G gene in oral and genital HPV infections needs further investigations, particularly among men.

## Supplementary Information

Below is the link to the electronic supplementary material.

Supplementary file1 (PDF 83 KB)

## Data Availability

The datasets analyzed during the current study are available from the study guarantor Karolina Louvanto on reasonable request.
