# Supplementary material for: The association of HLA-G polymorphism with oral and genital HPV infection in men
Source: Eur J Clin Microbiol Infect Dis. 2021 Oct 25;41(2):219–26. doi: 10.1007/s10096-021-04362-8 (PMC8770419; doi:10.1007/s10096-021-04362-8)
Supplement: Supplementary file 1 — Supplementary file1 (PDF 83 KB) [file 10096_2021_4362_MOESM1_ESM.pdf]

Table S1 Genotype specific HPV positivity at baseline in semen-, urethra- and oral samples among the 128 men of the Finnish Family HPV Study.

HPV genotype	semen n=86 (%)	urethra n=122 (%)	oral n=128 (%)
LR-HPV			
HPV6	7 (8.1)	7 (5.7)	1 (0.8)
HPV11	2 (2.3)	1 (0.8)	2 (1.6)
HPV43	…	1 (0.8)	1 (0.8)
total LR-HPV	9 (10.5)	9 (7.4)	4 (3.1)
HR-HPV			
HPV16	12 (14.0)	10 (8.2)	12 (9.4)
HPV18	3 (3.5)	…	1 (0.8)
HPV31	1 (1.2)	…	1 (0.8)
HPV33	4 (4.7)	5 (4.1)	4 (3.1)
HPV45	1 (1.2)	…	…
HPV51	…	1 (0.8)	…
HPV53	1 (1.2)	3 (2.5)	…
HPV56	…	2 (1.6)	…
HPV59	1 (1.2)	…	…
HPV66	2 (2.3)	1 (0.8)	…
HPV70	1 (1.2)	3 (2.5)	1 (0.8)
HPV82	…	…	5 (3.9)
total HR-HPV	23 (26.7)	20 (16.4)	21 (16.4)
any HPV+	27 (31.4)	27 (22.1)	24 (18.8)
HPV negative	59 (68.6)	95 (77.9)	104 (81.3)
multiple infection[1]	6 (7.0)	6 (4.9)	4 (3.1)

[1]Infection with two of more different HPV genotypes detected defined as multiple infection.
